# Gold (I) N-heterocyclic carbene complex inhibits mouse melanoma growth by p53 upregulation

**DOI:** 10.1186/1476-4598-13-57

**Published:** 2014-03-13

**Authors:** Abhishek Nandy, Sumit Kumar Dey, Sujata Das, Rudra Narayan Munda, Joydev Dinda, Krishna Das Saha

**Affiliations:** 1 Cancer Biology and Inflammatory Disorder division, CSIR-Indian Institute of Chemical Biology, Kolkata, West Bengal 700032, India; 2 Department of Biotechnology, North Orissa University, Sriram Chandra Vihar, Takatpur, Baripada, Mayurbhanj, Orissa 750003, India; 3 School of Physical Sciences, ITM University, Turari, Jhansi Road, Gwalior, MP 475001, India

**Keywords:** Gold (I) N-heterocyclic carbene complex, Growth inhibition, Apoptosis, Melanoma, Tumor

## Abstract

**Background:**

Cancer treatment using gold (I) complexes is becoming popular. In this study, a gold (I) N-heterocyclic complex designated as complex **3** was synthesized, its cytotoxicity was examined, and its anti-melanoma activity was evaluated *in vitro* and *in vivo*.

**Methods:**

Viability of cancer cells was determined by MTT assay upon treatment with various concentrations of a gold (I) N-heterocyclic carbene complex (complex **3**) in a dose and time dependent manner. Mouse melanoma cells B16F10 were selected for further apoptotic studies, including flowcytometric analysis of annexin binding, cell cycle arrest, intracellular ROS generation and loss in the mitochondrial membrane potential. ELISA based assays were done for caspase activities and western blots for determining the expression of various survival and apoptotic proteins. Immunocytology was performed to visualize the translocation of p53 to the nucleus. B16F10 cells were inoculated into mice and post tumor formation, complex **3** was administered. Immunohistology was performed to determine the expressions of p53, p21, NF-κB (p65 and p50), MMP-9 and VEGF. Student’s *t* test was used for determining statistical significance. The survival rate data were analyzed by Kaplan-Meier plots.

**Results:**

Complex **3** markedly inhibited the growth of HCT 116, HepG2, and A549, and induced apoptosis in B16F10 cells with nuclear condensation, DNA fragmentation, externalization of phosphatidylserine, activation of caspase 3 and caspase 9, PARP cleavage, downregulation of Bcl-2, upregulation of Bax, cytosolic cytochrome c elevation, ROS generation, and mitochondrial membrane potential loss indicating the involvement of an intrinsic mitochondrial death pathway. Further, upregulation of p53, p-p53 (ser 15) and p21 indicated the role of p53 in complex **3** mediated apoptosis. The complex reduced tumor size, and caused upregulation of p53 and p21 along with downregulation of NF-κB (p65 and p50), VEGF and MMP-9. These results suggest that it induced anti-melanoma effect *in vitro* and *in vivo* by modulating p53 and other apoptotic factors.

**Conclusions:**

The gold (I) N-heterocyclic carbene complex (C_22_H_26_N_6_AuO_2_PF_6_) designated as complex **3** induced ROS and p53 dependent apoptosis in B16F10 cells involving the mitochondrial death pathway along with suppression of melanoma tumor growth by regulating the levels of pro and anti apoptotic factors (p53, p21, NF-κB, VEGF and MMP-9).

## Background

The most widely used platinum-based anticancer drug cisplatin has several limitations like neurotoxicity, nephrotoxicity and development of resistance in some cancer cells [1]. In order to overcome these limitations, different metal complexes are being studied as anticancer agents. Among these, metal N- heterocyclic carbene (NHC) complexes are getting prominence as they readily fit the requirements for an efficient drug design [2]. NHC ligands are strong σ-donors as the free electron pairs of the nitrogen donate to the free p-orbital of the carbene, which reduces its π-backbonding capability [3]. This direct σ coordination of the carbene carbon with the gold (I) atom is responsible for its stability and biological activity. The strong metal-carbenic bond of the NHC complex contributes to the tight binding kinetics, resulting in lesser ligand dissociation [4]. Among various metal NHC complexes, silver NHC complexes have shown promise as anti-microbial agents [5], whereas gold (I) NHC complexes are reported to have anticancer activity on a number of cell lines [6]. There are a growing number of reports regarding the cytotoxicity of gold (I/III) NHC complexes against breast and prostate tumor cell lines [7].

The oxidation state I of gold has generated interest because it enables a more stable complex coordination which enhances its biological efficacy, the most popular example being that of gold (I) phosphine complex auranofin [8]. The complex, which was initially developed as an anti-rheumatic agent, recently had its potentiality as an anticancer agent well investigated [9]. As it is readily metabolized by thiol biomolecules, the coordinated ligands are mostly rendered impotent before the target enzyme is reached [10]. Therefore the development of more stable gold complexes with fine tuning of their ligands is of particular interest [11]. With the NHC ligands showing donor properties similar to those of phosphines, the ease of systemic modifications of the NHC substituents has made them attractive ligands for the development of gold complexes as potential apoptotic agents against cancer cells [12]. It may be mentioned that cancer cell death through apoptosis is a desired goal. Apoptosis is accompanied by chromatin condensation, DNA fragmentation, and phosphatidyl serine exposure to outer membrane, and may be dependent on ROS and p53 levels with the involvement of mitochondrial pathway or extrinsic pathway or both [13]. Tumour growth is governed by the levels of NF-κB, p53, VEGF, MMP-9 etc. [14,15].

We had earlier reported the cytotoxicity of a gold (I)-N-heterocyclic carbene complex with picoline functionalized benzimidazolin-2-ylidene ligand on HeLa (human cervical carcinoma), HepG2 (human hepatocellular carcinoma) and B16F10 (mouse melanoma) cell lines [16]. We have now synthesized and observed the cytotoxicity of another gold (I) NHC complex, designated as complex **3**, on a panel of four cancer cell lines including a highly metastatic mouse melanoma cell line, B16F10 [17]. Using the B16F10 cell line, we have examined the involvement of apoptosis after treatment of complex **3**. Next, the pathways of apoptosis including the role of ROS and p53 were explored. We have also determined the suppressive effect of complex **3** on B16F10 tumor growth in mice along with the status of pro- and anti-tumor survival factors. Our study shows that the gold complex **3** has potent anticancer activity towards mouse melanoma.

## Results

**Figure 1:**
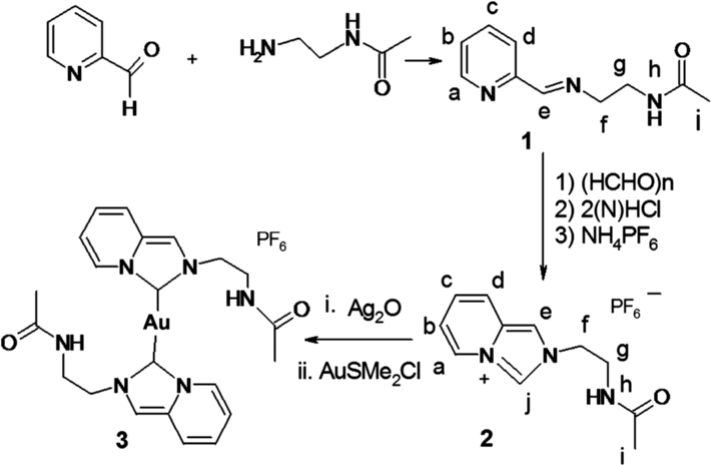
Synthesis of the gold(I) N-heterocyclic carbene complex 3.

The Schiff base was synthesized by the reported procedure [18] involving the reaction of pyridine 2-carboxaldehyde with N-(2-aminoethyl) acetamide in dry ethanol. The proligand **2** was synthesized from the corresponding Schiff base **1** by crusting with paraformaldehyde in dioxane followed by addition of dilute HCl in diethyl ether as described in a published procedure [19]. The layer separating from dioxane was taken out using a separating funnel and was mixed with minimum amount of water. Saturated solution of KPF_6_ was added to the aqueous chloride salt to get immediate precipitate of PF_6_ salt of **2**. The formation of imidazolium salt was confirmed by the appearance of imidazolium CH_2_ proton signal (9.55 ppm) in NMR. Signal for the proton bound to imidazolium-C2 appears at rather low field indicating its relatively high acidity. Au-carbene complex (**3)** formation [20] was supported by NMR spectroscopy; disappearance of the signal of the imidazolium-C2 proton in the ^1^HNMR spectrum and downfield shift (by 22.6 ppm) of the signal of carbenic carbon in ^13^C NMR spectrum in comparison to proligand confirmed the same. The carbenic carbon signal of **3** appears at 171.3 ppm (148.7 ppm for proligand) (Figure 1).

**Schiff base, 1** has ^1^H NMR (CD_3_CN, 25°C, 400 MHz): δ 7.89 (s, 1H, H^e^), 7.76 (d, J = 6.3 Hz, 1H, H^a^), 7.87 (d, J = 6.0 Hz, 1H, H^d^) 7.05 (m, 2H, H^b,c^), 5.03 (d, J = 6.0 Hz, 4H, H^f,g^), 3.33 (s, 1H, H^h^), 2.48(s, 3H, H^i^). ^13^C NMR (CD_3_CN, 100 MHz): δ 144.8, 139.0, 128.5, 123.8, 123.3, 117.4, 109.2, 32.2, 16.2. Anal. Calcd. For C_10_H_13_N_3_O (Additional file 1: Figure S1), C, 62.82; H, 6.81; N, 21.99; Found C, 62.58; H, 6.66; N, 21.89%.

**Proligand, 2** has ^1^H NMR (CD_3_CN, 25°C, 400 MHz): δ 9.55 (s, 1H, H^j^), 8.60 (d, J = 6.0 Hz, 1H, H^a^), 8.10 (s, 1H, H^e^), 7.87 (d, J = 6.0 Hz, 1H, H^d^) 7.25 (m, 2H, H^b,c^), 5.07 (d, J = 6.0 Hz, 4H, H^f,g^), 3.33 (s, 1H, H^h^), 2.48 (s, 3H, H^i^). ^13^C NMR (CD_3_CN, 100 MHz): δ 148.7, 145.7, 139.9, 129.2, 124.2, 123.6, 117.8, 109.4, 32.6, 32.8, 16.4 (Additional file 2: Figure S2). Anal. Calcd. For C_11_H_14_N_3_OPF_6_, (Additional file 3: Figure S3) C, 37.82; H, 4.01; N, 12.03; Found C, 37.58; H, 4.03; N, 11.92%.

**Gold-Complex, 3** has ^1^H NMR (DMSO-d_6_, 400 MHz, 30°C): δ 8.42 (d, J = 6.9 Hz, 1H, H^a^), 7.81 (s, 1H, H^e^), 7.59 (d, J = 8.1 Hz, 1H, H^i^), 7.02 (t, J = 7.3 Hz, 1H, H^c^), 6.79 (t, J = 8.1 Hz, 1H, H^b^), 5.15 (s, 4H, H^f,g^), 3.34 (s, 1H, H^h^), 2.49 (s, 3H, H^i^). ^13^C NMR (DMSO-d_6_, 100 MHz, 35°C) δ 171.3, 149.5, 141.4, 129.6, 124.7, 124.0, 118.3, 109.8, 32.8, 32.9, 15.9 (Additional file 4: Figure S4). Anal. Calcd. For C_22_H_26_N_6_AuO_2_PF_6_, (Additional file 5: Figure S5) C, 35.29; H, 3.47; N, 11.23; Found C, 35.16; H, 3.42; N, 11.18%.

### Complex 3 induces apoptosis in B16F10 cells

**Figure 2:**
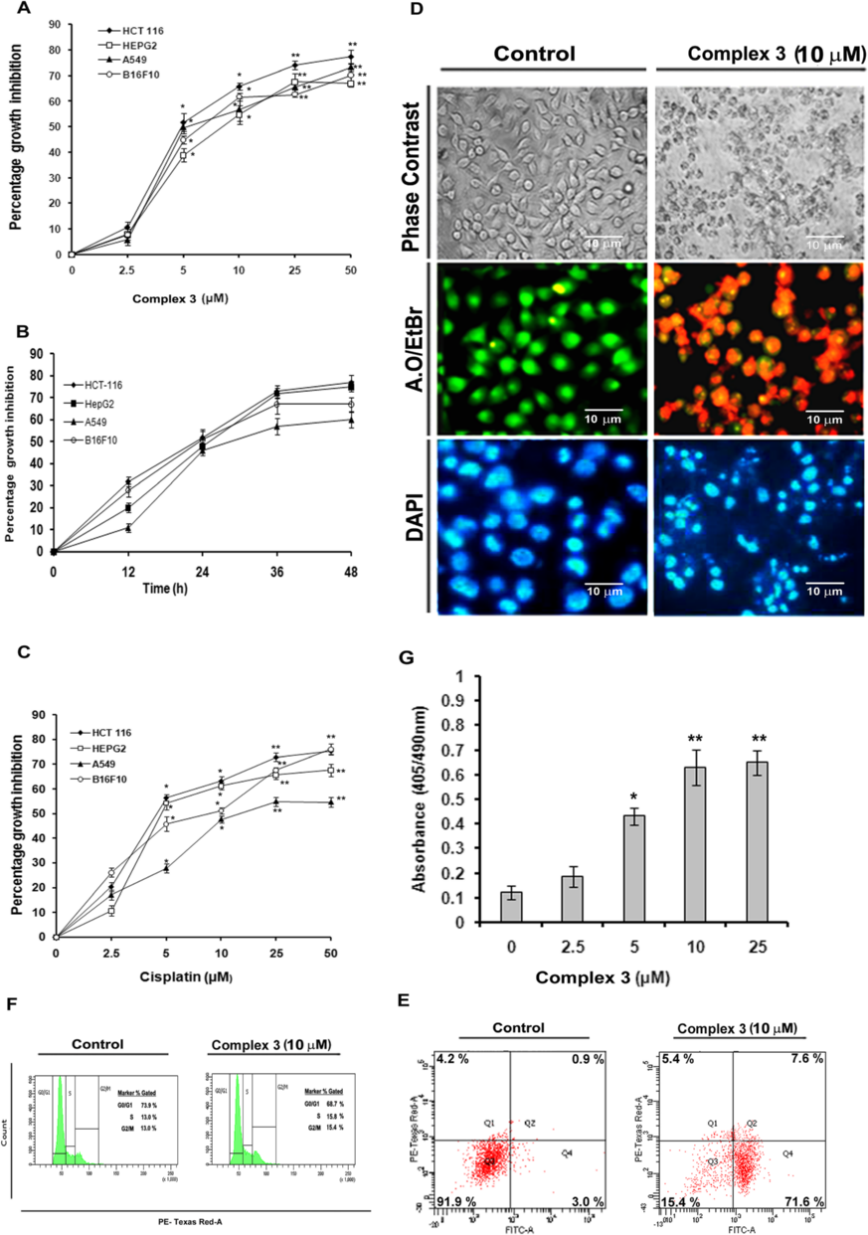
**Growth inhibition and the subsequent evaluation of apoptosis of cancer cells in presence of complex 3. A**, Percentage growth inhibition of HCT 116, HepG2, A549 and B16F10, following treatment with complex **3** (0, 2.5, 5,10 and 25 μM) for 24 h. **B**, Percentage of growth inhibition of HCT-116, HepG2, A549 and B16F10, following treatment with GI_50_ concentration of complex **3** for 0, 12, 24 and 48 h. **C**, Percentage of growth inhibition of HCT-116, HepG2, A549 and B16F10, following treatment with cisplatin (0, 2.5, 5,10 and 25 μM) for 24 h. **D**, Morphological images of the B16F10 cells treated with complex **3** (0 and 10 μM) in the upper row. Acridine orange/EtBr stained images of B16F10 cells treated with complex **3** (0 and 10 μM) in the middle row. DAPI stained image of the B16F10 cells treated with complex **3** (0 and 10 μM) in the lower row. Scale Bar = 10 mm. Magnification at 40×. **E**, Flow cytometric analysis of apoptosis induction in B16F10 cells treated with complex **3** (0 and 10 μM) for 24 h. **F**, Cell cycle analysis of complex **3** (0 and 10 μM) treated B16F10 cells for 24 h. **G**, DNA fragmentation analysis of complex **3** (0, 2.5, 5, 10 and 25 μM) treated B16F10 cells for 24 h. Values are mean ± S.D and represent one of the 3 representative experiments. *P < 0.05, **P < 0.01 and ***P < 0.001.

The cytotoxic effect of complex **3** was investigated on HCT 116, HepG2, A549 and B16F10 cells by MTT assay. Treatment of different concentrations of complex **3** (0 to 50 μM) reduced the viability of the cancer cells in a dose dependent manner (Figure 2A) in different degrees, with higher toxicity in HCT 116 (GI_50_ 4.73 ± 0.20 μM) after 24 h. It also showed good cytotoxic activity on the other three cell lines, HepG2 (GI_50_ 9.48 ± 0.25 μM), B16F10 (GI_50_ 9.4 ± 0.26 μM) and A549 (GI_50_ 13.71 ± 0.15 μM) after 24 h. In a time dependent study, complex **3** (GI_50_ concentrations) induced 77% and 75% mortality in HCT 116 and HepG2 cells respectively, 67% mortality in B16F10 cells and 60% mortality in A549 cells after 48 h (Figure 2B and Additional file 6: Table S2). The commonly used anticancer drug cisplatin had a marginally higher rate of cytotoxicity as compared to **3** after 24 h (Figure 2C and Additional file 6: Table S1). However, the proligand **2** failed to elicit any cytotoxicity in the cancer cell lines mentioned in this study. Thus, complex **3** is a good cytotoxic agent against the cancer cells used in this study.

B16F10 is a highly metastatic form of a mouse melanoma cell line [17]. Complex **3** showed significant cytotoxicity towards this cell line like the other cancer cell lines used in this study. Therefore B16F10 cells were used as model for further study on cytotoxicity induced by complex **3**. Cancer cell death through apoptosis is a desirable goal. Complex **3** showed characteristic apoptotic changes in morphology like cell shrinkage and rounding (Figure 2D, upper panel), and nuclear changes such as chromatin condensation and DNA fragmentation, seen using the nuclear staining dye AO along with EtBr and DAPI (Figure 2D, middle panel and lower panel respectively). The number of condensed bright fragmented nuclei seen in complex **3** treated cells after DAPI staining was higher, while yellowish, orange and red colored nuclei or a combination of these colored nuclei were observed upon staining with AO/EtBr. Marked increase in the number of DNA fragments in complex **3** treated cells as seen by using the DNA fragmentation assay kit (Figure 2G) also corroborated the findings in Figure 2D. Thus, cytotoxicity induced by complex **3** in B16F10 cells may involve apoptosis.

Phosphatidylserine [PS] externalization from inner cell membrane to outer membrane is a prerequisite for apoptosis. Externalized PS can bind with annexin V [21]. B16F10 cells exposed to complex **3** showed higher percentage of annexin V-FITC binding cells after staining with annexin V-FITC and PI. After 24 h of treatment with 10 μM of complex **3**, percentage of apoptotic cells was 79.2% as compared to 3.9% in the vehicle treated cells (Figure 2E). These findings suggested that complex **3** induced death of B16F10 cells resulting from apoptosis.

Cell cycle arrest at various phases of cell division precedes apoptosis [13]. There was a gradual decrease in the number of cells in G1 phase upon treatment with complex **3** (0 and 10 μM) for 24 h (Figure 2F). The percentage of G0–G1 population for treated cells was 68.7% at 24 h as compared to 73.9% for vehicle treated cells, indicating a decrease of 5.2% of cell population in the G0–G1 phase. The percentages of S phase population for such cells treated with complex **3** (0 and 10 μM) were 13.0% and 15.8% respectively indicating an increase in 2.8% of cell population in the S phase after 24 h. Moreover, the percentages of G2-M phase population for complex 3 (0 and 10 μM) treated B16F10 cells at 24 h were 13.0% and 15.4% respectively indicating an increase of 2.4% of cell population in the G2-M phase. This indicated that complex **3** may mediate cell cycle arrest at the G2-M phase.

### Complex 3 induced apoptosis of B16F10 cells proceeds via the mitochondrial death pathway involving ROS

**Figure 3:**
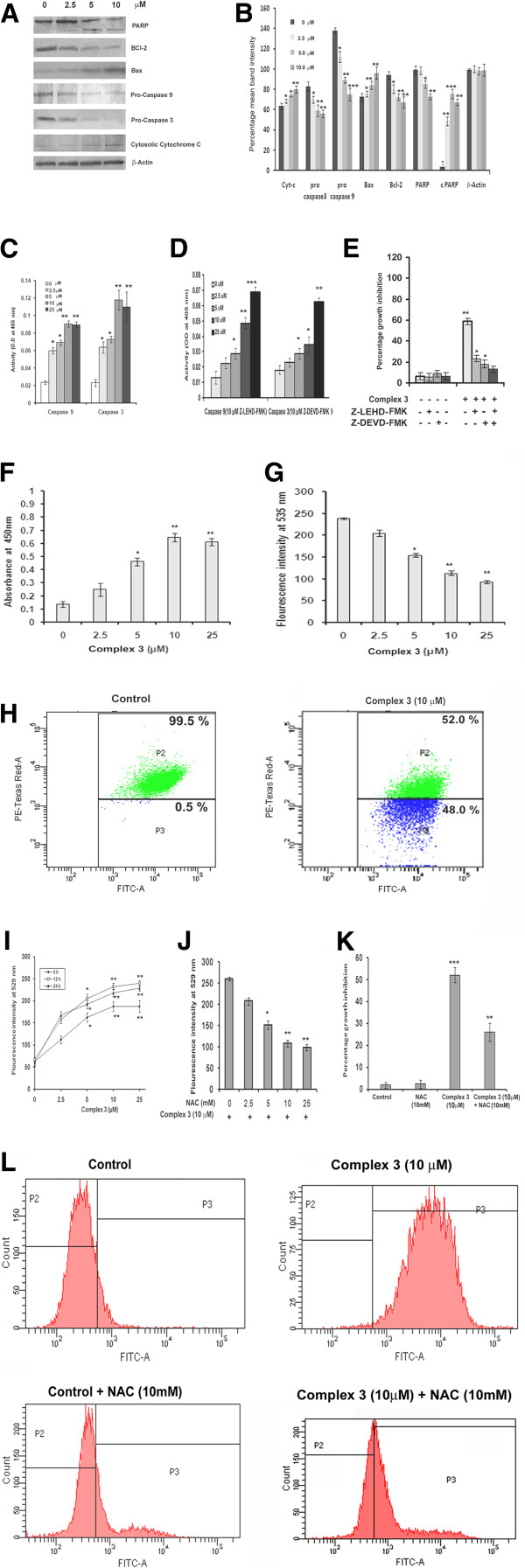
**Elucidation of the ROS mediated, mitochondrial death pathway upon caspase activation. A**, Expression of various apoptotic proteins following treatment of cells with complex **3** for 24 h, with β-Actin as loading control. **B**, Densitometric analysis of proteins detected by Western blot. **C**, Determination of caspase 9 and 3 activities at 405 nm, following treatment of cells with complex **3** for 24 h. **D**, Determination of caspase 9 and 3 activities at 405 nm in cells, incubated 1 h with Z-LEHD-FMK and Z-DEVD-FMK prior to treatment with complex **3** for 24 h. **E**, Percentage of growth inhibition of cells following incubation with caspase inhibitors Z-LEHD-CHO and Z-DEVD-CHO (for caspase 9 and caspase 3 respectively), along with complex **3** for 24 h. **F**, Cytochrome c release into the cytosol after treatment of cells with complex **3** as determined at 450 nm assay. **G**, Loss of ∆ψm of complex **3** treated cells after 24 h. **H**, Flowcytometric analysis of loss of ∆ψm in presence of complex **3** after 24 h. **I**, ROS generation upon treatment of complex **3** for 6, 12 and 24 h at 529 nm. **J**, ROS generated in cells treated with complex **3** and with varying concentrations of NAC (0.2, 1, 5 and 10 μM) after 24 h at 529 nm. **K**, Percentage growth inhibition of cells in presence or absence of either complex **3** or NAC or in presence of both of them. **L**, Flowcytometric analysis ROS generation in cells following treatment with complex **3** along with NAC after 24 h. Values are mean ± S.D and represent one of the 3 representative experiments. *P < 0.05, **P < 0.01 and ***P < 0.001.

“Apoptotic trigger” is initiated when the ratio of the pro- and anti-apoptotic members of the Bcl-2 family is unbalanced. Downregulation of the anti-apoptotic Bcl-2 protein and binding of pro-apoptotic Bax protein to the mitochondria membrane cause disruption of the mitochondrial membrane leading to the release of cytochrome c from mitochondria to cytosol. These events lead to the activation of caspase 9 (initiator caspase) and caspase 3 (executioner caspase). Activated caspases induce the cleavage of PARP, thereby perturbing its ability for DNA repair and leading to cell death [13]. Treatment of complex **3** suppressed the level of Bcl-2, activated Bax, lowered the expressions of pro-caspase-3 and pro-caspase-9, promoted PARP cleavage, and increased the level of cytosolic cytochrome c in a dose dependent manner (Figure 3A). Densitometric analyses of the western blots have been shown in Figure 3B. Status of active caspase-3 and caspase-9, which increased with doses of complex **3** as seen in the study using assay kit (Figure 3C), were dampened upon pre-incubation of cells with Z-LEHD-FMK and Z-DEVD-FMK (Figure 3D). Moreover cells pre-incubated with Z-LEHD-FMK and Z-DEVD-FMK exhibited higher cell viability as compared to cells treated with either one or none of these inhibitors prior to treatment with complex **3** (Figure 3E). Elevated level of cytosolic cytochrome c was also observed (Figure 3F) 24 h after complex **3** treatment (0 and 10 μM), along with a dose dependent decrease in ∆Ψm (Figure 3G). Moreover, flowcytometric analysis of vehicle treated cells revealed that 99.5% of the cell population exhibited fluorescence at the PE-Texas Red A channel indicating a higher level of cells having a healthy ∆Ψm, whereas complex **3** (10 μM) treated cells revealed that 52.0% of the cell population fluorescence at the PE-Texas Red A channel, hinting at a loss of ∆Ψm in 47.5% of cell population after 24 h (Figure 3H). These results indicate that complex **3** may induce apoptosis via the mitochondrial pathway.

Generation of reactive oxygen species (ROS) is a key factor of apoptotic cell death. The maximum ROS generation was exhibited at 12 h after treatment of cells with complex **3** (Figure 3I). Treatment with NAC (N-acetyl-cysteine) 1 h prior to complex **3** treatment resulted in decrease in ROS production (Figure 3J) along with inhibition of cell death (Figure 3K). Also, flowcytometric analysis revealed that the FITC mean intensity was 410 in vehicle treated cells but 8829 in complex **3** (10 μM) treated cells after 24 h, indicating a shift in FITC mean intensity from the vehicle treated cells to the cells treated with complex **3** (10 μM) (Figure 3L). However, incubation with NAC 1 h prior to complex **3** (0 and 10 μM) treatment resulted in a mean FITC mean intensity of 956 for vehicle treated cells but 1928 for **3** (10 μM) treated cells after 24 h, indicating a shift in intensity from the vehicle treated cells to the cells treated with complex **3** (10 μM) (Figure 3L). The study demonstrates that complex **3** induced apoptosis in B16F10 may proceed via the ROS mediated pathway.

### B16F10 cell death triggered by complex 3 results in p53 activation in ROS dependent manner

**Figure 4:**
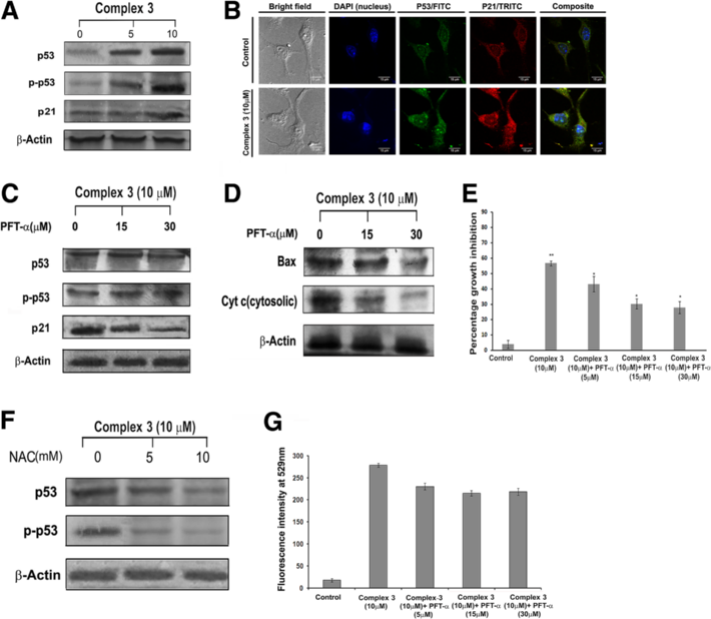
**Evaluation of the status of p53 following treatment of cells with complex 3. A**, Upregulation of p53, p-p53 (ser 15) and p21. **B**, Upregulation of p53, p21 and subsequent nuclear translocation of p53 upon treatment of cells with complex **3** (0 and 10 μM). Scale Bar = 15 mm. Magnification at 40×. **C**-**D**, Expression profile of p53 and p-p53 (ser 15) along with the downregulation of p21, Bax and cytosolic cytochrome c upon treatment with 15 and 30 μM of pifthrin-α in presence of complex **3** (10 μM) after 24 h. β-Actin was used as a loading control. **E**, Percentage growth inhibition of cells in presence of pifthrin-α (0, 5, 15 and 30 μM) and complex **3** (10 μM) with respect to the control (cells without treatment with complex **3**). **F**, Down regulation of p53 and p-p53 (ser 15) in the presence of NAC. β-Actin was used as a loading control. **G**, ROS generation as determined at 529 nm of cells treated with complex **3** (10 μM) in the presence of PFT-α (0, 5, 15, 30 μM). Values are mean ± S.D and represent one of the 3 representative experiments. *P < 0.05 and **P < 0.01.

Apoptosis is generally regulated by p53. Therefore, the role of complex **3** on p53 activation was examined. Treatment of B16F10 cells with complex **3** (0, 5 and 10 μM) resulted in upregulation of p53, p21 and phospho-p53 (p-p53) (Figure 4A). Treatment with 10 μM of complex **3** for 24 h resulted in increased translocation of p53 to the nucleus along with increased expression of p21 as compared to the untreated cells (Figure 4B). The corresponding fluorescence intensity graphs have been provided in a supplementary figure (Additional file 7: Figure S6). Pretreatment of pifthrin-α (PFT-α), an inhibitor of p53 transactivation, inhibited the expression of p21 along with reduction in the level of p53 dependent proteins such as Bax and cytosolic cytochrome c. However, PFT-α failed to downregulate the expression of p-p53 (ser 15) whereas p53 registered a slight reduction in its expression (Figure 4C-D). Also, pretreatment with PFT-α prevented growth inhibition of cells treated with complex **3** (Figure 4E). Therefore p53 may play a significant role in complex **3** mediated apoptosis of B16F10 cells.

Treatment of B16F10 cells with NAC 1 h prior to complex **3** addition led to the inhibition of p53 as well as p-p53 (phosphorylated at ser-15) (Figure 4F). However, treatment of PFT-α failed to suppress ROS production (Figure 4G), indicating that p53 may not be involved in ROS generation whereas ROS maybe involved in p-p53 up regulation.

### Complex 3 exhibits antitumor activity in B16F10 melanoma model

**Figure 5:**
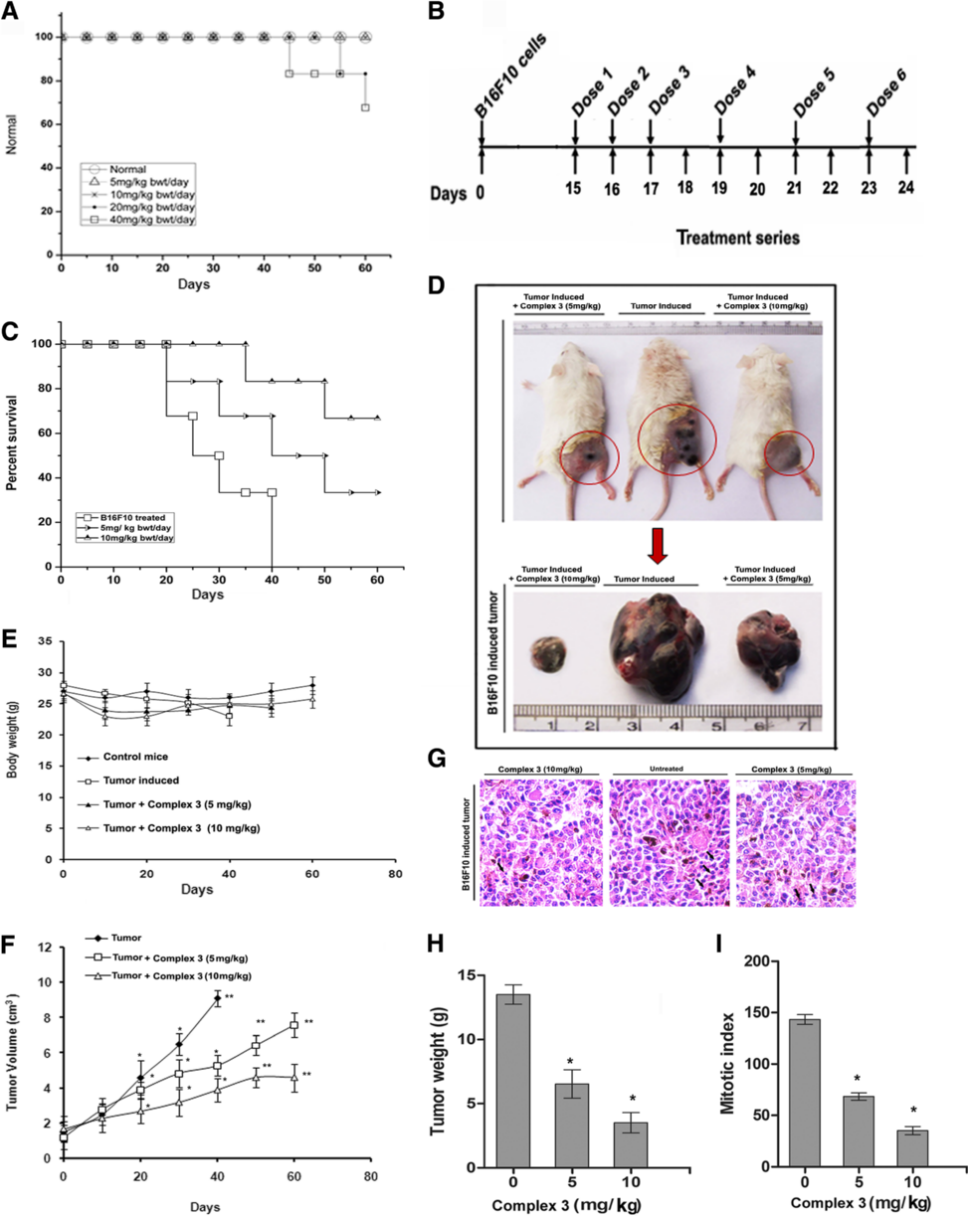
**B16F10 cell induced tumor growth in BALB/c mice in the presence or absence of complex 3. A**, Mice toxicity survival curve upon administration of various doses of complex **3**. **B**, Treatment series depicting the sequence followed for complex **3** administration, following tumor growth. **C**, Percentage survival of tumor carrying mice upon administration of complex **3** (0, 5, 10 mg/kg body weight of mice) over a period of 60 days. **D**, Tumor size variation upon administration of complex **3** (0, 5, 10 mg/kg body weight of mice). **E**-**F**, body weight (g) and tumor volume (cm^3^) of mice administered with complex **3** (0, 5 and 10 mg/kg weight of mice). **G**-**I**, H/E staining (→ depicting the intact and fragmented nuclei in the control and the treated tumor sections respectively), tumor weight (g) and mitotic index of tumors excised from mice administered with complex **3** (0, 5 and 10 mg/kg body weight). The survival rate data were analyzed by Kaplan-Meier plots. Values are mean ± S.D and represent one of the 3 representative experiments . *P < 0.05 and **P < 0.01.

Complex **3** administered intraperitoneally at the dosages of 5, 10, 20, and 40 mg/kg body weight per day till the 60^th^ day showed 100% survivability for dosages of 0, 5 and 10 mg/kg, 80% survivability with 20 mg/kg, and 70% survivability with 40 mg/kg body weight respectively (Figure 5A). Administration of complex **3** (0, 5 and 10 mg/kg body weight/day) was started from 15^th^ day onward since the day of injection of B16F10 cells in the mice and was continued till the 23^rd^ day. The mice were sacrificed on the 24^th^ day (Figure 5B).

Mice bearing the B16F10 tumor but not administered complex **3** faced total mortality by the 40^th^ day, whereas treatment with 5 and 10 mg/kg mouse body weight of the complex ensured 30% and 70% survivability till the 60^th^ day (Figure 5C). Dose dependent decrease in the tumor size was observed following treatment with complex **3** (0, 5 and 10 mg/kg of mouse body weight) (Figure 5D). Haematoxylin and Eosin staining revealed an increase in number of fragmented nuclei in a dose dependent manner (Figure 5G). Both mitotic index (an indication of cell proliferation) and tumor weight decreased upon treatment with complex **3** (Figure 5H-I). Body weight remained nearly constant (Figure 5E), but the tumor volume decreased in a dose dependent manner (Figure 5F). The results show that complex **3** is a potent suppressor of melanoma tumor.

### Translocation and localization of p53/p21, NF-kB p50/p65, VEGF and MMP-9 proteins in tumor as determined by immunohistological analysis

**Figure 6:**
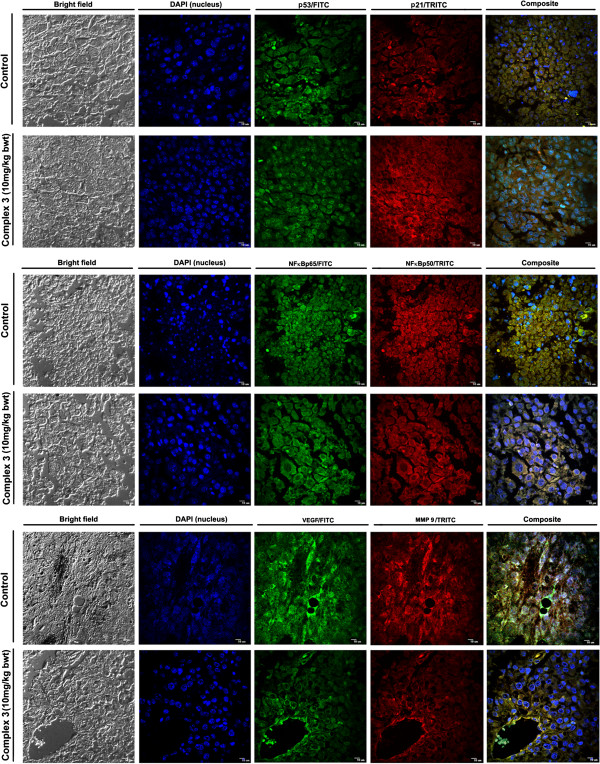
**The expression profile of tumor promoting and inhibiting proteins of tumor sections excised out from tumor bearing mice administered with complex 3 (0 and 10 mg/kg/body weight of mice).** Immunohistochemical analysis of p53/p21 (upper panel), NF-қB p65/p50 subunits (middle panel), and VEGF/MMP9 (lower panel). Magnification at 20×. Scale Bar = 15 mm.

The p53 protein expression and translocation to the nucleus was upregulated along with the upregulation of p21 in the tumor sections of the mice administered with complex **3** (10 mg/kg body weight of mice) with respect to the tumors sections of the mice not administered complex **3** (Figure 6, upper panel). The translocation of the anti-apoptotic NF-κB protein (p65 and p50) was inhibited upon administration of complex **3** (10 mg/kg body weight of mice) (Figure 6, middle panel). Following treatment with complex **3** (10 mg/kg body weight of mice), the expressions of angiogenic and metastatic markers such as VEGF and MMP-9 respectively were also inhibited (Figure 6, lower panel), indicating that tumor death occurs by activation of p53 and inhibition of the NF-κB protein, VEGF and MMP-9. The corresponding fluorescence intensity graphs have been provided as a supplementary figure (Additional file 8: Figure S7).

## Discussion

Our study revealed that complex **3** showed appreciable cytotoxicity towards a panel of four cancer cell lines (HCT 116, HepG2, A549 and B16F10) after 24 h, but in different degrees, with the GI_50_ being comparable to that of cisplatin. Interestingly, 15 μM of cisplatin was toxic to the peripheral mononuclear blood cells (PBMCs), whereas complex **3** up to 80 μM did not show any toxicity (Additional file 6: Table S3). This was in accordance with earlier reports revealing the low cytotoxicity of gold and silver N-heterocyclic carbene complexes towards non transformed cell lines with respect to cisplatin [22]. Earlier, Rubbiani *et al.* and Wang *et al.* had reported the efficacy of other gold (I) NHC complexes on MCF-7, HT-29, HepG2 and U-87MG which resulted in increased AnnexinV-FITC binding, ROS generation, loss of ΔΨm, along with elevation of Bax, p53, p-p53 (ser 15), p21, cleaved PARP and cleaved caspase 3 [4,23], thereby supporting the results obtained following treatment of B16F10 with complex **3**.

Moreover, inhibition of caspase 9 and caspase 3 with Z-LEHD-FMK (caspase 9 inhibitor) and Z-DEVD-FMK (caspase 3 inhibitor) generally decreased the growth inhibitory potential of complex **3**. This indicates that complex **3** may initiate apoptosis via the mitochondrial death pathway.

The role of p53 is quite essential here. The inhibition of p53 transactivation by pifthrin-α (PFT-α) led to a down regulation of its transcriptional targets such as p21 and Bax. p-p53 (ser 15) is a marker for DNA damage, occurring mainly due to excessive ROS generation. However PFT-α failed to inhibit the expression of p-p53, thereby indicating that ROS acted upstream of p53 following complex **3** treatment. However, p21 and Bax being the transcriptional targets of p53 were affected by the inhibitory role of PFT-α on p53 as a transcription factor. As Bax translocation to the mitochondria results in the release of cytochrome c in the cytosol, inhibition of Bax expression inhibited the release of cytochrome c into the cytosol. Also treatment with PFT-α, 1 h prior to treatment with complex **3** did not induce growth inhibition. This implies that complex **3** may induce apoptosis by involvement of p53.

Generation of ROS upon induction of apoptosis by gold (I) NHC complex has been already reported [4]. When cells were pre-incubated with NAC (a ROS scavenger), there was an increase in cell viability in presence of complex **3**, along with down regulation of p53 and p-p53 (ser 15). However, pre-incubation with PFT-α did not prevent ROS generation. Thereby, complex **3** may induce ROS generation upstream of p53 and induce apoptosis via ROS mediated pathway. Therefore, we may conclude that complex **3** mediates apoptosis in B16F10 cells via a ROS mediated mitochondrial death pathway involving p53 up regulation.

One suggested mode of anticancer activity of gold (I)-NHC complexes is by accumulation in the mitochondria leading to ΔΨm perturbations and by the prevention of the catalytic activity of the selenoenzyme thioredoxin reductase (TrxR), which in turn induces extensive oxidation of thioredoxins (Trxs) [24]. In the light of this report, we suggest that complex **3** may induce apoptosis in B16F10 cells by mitochondrial accumulation and inhibition of the catalytic activity of thioredoxin reductase.

Yan *et al.* had reported that a cyclometalated gold (III) complex with an N-heterocyclic carbene ligand induced suppression of PLC tumor in mice at a dosage of 10 mg/kg body weight [25]. Similarly we observed that treatment of male BALB/c mice bearing B16F10 tumor with complex **3** (5 and 10 mg/kg of mice body weight) induced decrease in tumor size, volume, and weight as well as in mitotic index, with an increase in fragmented nuclei in a dose dependent manner with respect to the control without any deleterious effect on the health of the animal. Treatment of such mice with complex **3** (10 mg/kg body weight) showed up regulation of p53 and p21 expression, which was also observed in case of B16F10 cells treated in vitro by complex **3**. These findings indicate that complex **3** may mediate B16F10 cell growth inhibition via up regulation of p53 and p21. Auranofin has been reported to down regulate NF-κB and VEGF [26,27]. Administration of complex **3** (5 mg/kg body weight of mice) resulted in down-regulation of NF-κB along with VEGF and MMP-9. Our results thereby indicate that the antitumor activity of complex **3** may be mediated by upregulation of p53 and p21, and downregulation of NF-κB p65 and p50 subunits along with VEGF and MMP-9.

The gold complex induced ROS and p53 dependent apoptosis in B16F10 cells involves the mitochondrial death pathway. Complex **3** suppressed melanoma tumor growth by regulating the level of pro anti apoptotic factors (p53, p21, NF-κB, VEGF and MMP-9). Also, it was cytotoxic on three cell lines like HCT 116, HepG2 and A549 examined in our study. So it may be a good anticancer agent at least towards some other cancer cells and tumors.

## Materials and methods

### Reagents

Dulbecco’s modified Eagle medium (DMEM), fetal bovine serum (FBS), penicillin, streptomycin, neomycin (PSN) antibiotic, trypsin and ethylene di-amine tetra-acetic acid (EDTA) were obtained from Gibco BRL (Grand Island, NY, USA). Tissue culture plastic wares were obtained from NUNC (Roskilde, Denmark). Antibodies were obtained from Santa Cruz Biotechnology (Santa Cruz, CA). Organic solvents used were of HPLC grade. All other chemicals including doxorubicin, NAC used were from Sigma Chem. Co. (St. Louis, MO, USA) or mentioned otherwise.

### Synthesis of the Schiff base 2-pyridyl-N-(2-ethylacetamido) methylamine (1)

Pyridine-2-carboxaldehyde (450 mg, 4.21 mmol) and N-acetylethylenediamine (429 mg, 4.21 mmol) were taken in dry ethanol (25 ml) and refluxed under stirring condition for 6 h. Volatiles were removed under reduced pressure and dried under vacuum. Analytically pure samples were obtained after crystallization by slow diffusion of diethyl ether into chloroform solution of Schiff base. The solid mass was dried in vacuum and the yield was 68% (547 mg, 2.86 mmol).

### Synthesis of proligand 2-pyridin- N-(2-ethylacetylamido)-2-yl-2H-imidazo [1, 5-a] pyridin-4-ylium hexafluorophosphate (2)

2-Pyridyl-N-(2-ethylacetoamido) methylamine **1** (450 mg, 2.36 mmol) and crushed 91% paraformaldehyde powder (75.6 mg, 2.70 mmol) were taken in 20 ml of dioxane and stirred for 4 h to form a slurry; then the mixture was refluxed for 10 h. A 5 ml aliquot of 2 (N) HCl in diethyl ether was added slowly to the cold slurry, resulting in immediate separation of layers - a lower layer of a yellowish red viscous liquid and a colorless liquid in the upper part. Then 5 ml water was added to the solution and the aqueous phase was separated by a separating funnel. Excess saturated aqueous KPF_6_ was added to the solution and immediately a white precipitate was obtained. This precipitate was then filtered out and recrystallized from acetonitrile and diethyl ether. The resultant solid mass was dried in vacuum and the yield was 66% (542 mg, 1.55 mmol).

### Preparation of gold (I) N- heterocyclic carbene complex 3

Proligand **2** (200 mg, 0.57 mmol) and silver oxide (67.2 mg, 0.29 mmol) were dissolved in acetonitrile (15 ml) and the mixture was stirred for 5 h. The volume of the solution containing a silver carbene complex was reduced to 5 ml. In 5 ml acetonitrile solution of AuSMe_2_Cl (85.4 mg, 0.29 mmol), the silver carbene complex was added drop wise, and immediately a white precipitate was observed. The mixture was filtered; the volatiles were removed under reduced pressure and dried under vacuum. Analytically pure sample of a gold (I) carbene complex **3** was obtained after crystallization by slow diffusion of diethyl ether into an acetonitrile solution of complex **3**. Yield was 80% (173.5 mg, 0.23 mmol).

### Cell culture

Cell lines, such as HCT-116 (human colorectal carcinoma), HepG2 (human hepatocellular carcinoma), A549 (human non small lung carcinoma) and B16F10 (mouse melanoma), were obtained from National Centre for Cell Science, Pune, India. These cell lines were cultured in DMEM supplemented with 10% FBS and 1% antibiotic (PSN) and incubated at 37°C in a humidified atmosphere with 5% CO_2_. After achieving 75–80% confluence, cells were harvested with 0.025% trypsin and 0.52 mM EDTA in phosphate buffered saline (PBS) and were seeded at desired density to allow them to re-equilibrate a day before the start of experimentation. All experiments were conducted in DMEM supplemented with 10% FBS and 1% antibiotic (PSN) solution.

### Cell viability assay

Cells were treated with various concentrations of complex **3** (0, 2.5, 5, 10, 25 and 50 μM) dissolved in 0.05% of DMSO for 24 h and their respective GI_50_s were determined. Moreover, viability of cells treated with the GI_50_ concentrations of complex **3** (determined after 24 h) for 12, 24, 36 and 48 h were determined by MTT assay as described earlier [28]. In another set of experiments, cells treated with cisplatin (0, 2.5, 5, 10, 25 and 50 μM) for 24 h were taken as positive control. Absorbance of the solubilized intracellular formazan was measured at 595 nm using an ELISA reader (Model: Emax, Molecular device, USA).

### Assessment of cellular death parameters under a microscope

B16F10 cells treated with complex **3** (0 and 10 μM) for 24 h were viewed under a phase contrast microscope. Cells were stained with DAPI (4′, 6-diamidino-2-phenylindole) for the detection of chromatin condensation and DNA fragmentations. Cells were stained with acridine orange (A/O) and ethidium bromide (EtBr) to distinguish between live and apoptotic death. Cells were observed under an inverted phase contrast/fluorescent microscope (Model: OLYMPUS IX70, Olympus Optical Co. Ltd., Shibuya-ku, Tokyo, Japan) and images were acquired.

### Detection of apoptosis using flow cytometry

Apoptosis was assayed by using an annexin V-FITC apoptosis detection kit (Calbiochem, La Jolla, CA) as described earlier [21]. B16F10 cells treated with complex **3** (0 and 10 μM) for 24 h were stained with PI and annexin V-FITC according to manufacturer’s instructions. The percentage of live, apoptotic and necrotic cells were analyzed by BD LSRFortessa cell analyzer (Becton Dickinson, San Jose, CA, USA). Data from 10^6^ cells were analyzed for each sample.

### Analysis of DNA fragmentation by ELISA

B16F10 cells were treated with complex **3** (0, 2.5, 5, 10 and 25 μM) for 24 h and fragmented DNA was assayed by an ELISA based method as earlier described [13].

### Analysis of cell cycle arrest

Cell cycle arrest was analyzed by treating cells with complex **3** (0 and 10 μM) for 24 h followed by PI staining, as described earlier [13]. The percentages of cell population undergoing cell cycle arrest at various stages of mitosis were analyzed by BD LSRFortessa cell analyzer (Becton Dickinson, San Jose, CA, USA). Data from 10^6^ cells were analyzed for each sample.

### Caspase-3 and 9 activity assay

B16F10 cells were treated with complex **3** (0, 2.5, 5, 10 and 25 μM) for 24 h and caspase-3 and caspase-9 activities were quantified with commercially available caspase-3/CPP32 and caspase-9 colorimetric Assay kit (BioVision Research Products, Mountain View, CA) as described earlier [13]. Caspase activity was spectrophotometrically detected at 405 nm on an ELISA reader (Model: Emax, Molecular device, USA). In another set of experiments, B16F10 cells were treated with 10 μM of Z-DEVD-FMK (caspase-3 inhibitor) and Z-LEHD-FMK (caspase-9 inhibitor) 1 h prior to treatment with complex **3** (0 and 10 μM) and MTT assay was done to evaluate growth inhibition of cells.

### Measurement of intra cellular ROS level upon treatment of complex 3 on B16F10 cells

For the detection of intracellular ROS generation, B16F10 cells treated with complex **3** (0 and 10 μM) for 24 h were incubated with 10 μM of H2DCFH-DA (2′, 7′-dichlorofluorescein diacetate, Molecular Probes) with or without NAC (N-acetyl-cysteine, added 1 h prior to complex **3** treatment) for 25 min at 37˚C, following which cells were analyzed by BD LSRFortessa cell analyzer. Data from 10^6^ cells were analyzed for each sample as described earlier [13]. In another set of experiments, B16F10 cells treated with complex **3** (0, 2.5, 5, 10 and 25 μM) for 6, 12 and 24 h were incubated with 10 μM of H2DCFH-DA for 25 min at 37°C. The increase in fluorescence due to production of ROS was noted (excitation 488 nm, emission 529 nm) using a fluorimeter. In another set of experiment, cells were incubated with NAC (0, 2.5, 5, 10 and 25 mM), 1 h prior to treatment with complex **3** (10 μM) for 24 h and the fluorescence increase because of production of ROS was noted (excitation 488 nm, emission 529 nm) using a fluorimeter. Further, MTT assay was done on cells incubated with NAC (0 and 10 mM) prior to addition of complex **3** (0 and 10 μM) for 24 h.

### Measurement of mitochondrial membrane potential

To measure mitochondrial membrane potential (MMP), B16F10 cells treated with complex **3** (0 and 10 μM) for 24 h were incubated with JC-1(5,5′,6,6′-tetrachloro-1, 1′, 3, 3′-tetraethylbenzimidazolylcarbocyanine iodide, Sigma). The reagent gives fluorescence in the FITC channel for green monomers in case of healthy cells having high mitochondrial membrane potential and in the PE-Texas Red A channel for red aggregates signifying apoptotic cells indicating a drop in the mitochondrial membrane potential. Data from 10^6^ cells were analyzed in a BD LSRFortessa cell analyzer for each sample.

Moreover, B16F10 cells treated with complex **3** (0, 2.5, 5, 10 and 25 μM) for 24 h were incubated with Rhodamine 123 and the fluorescence emission was measured in a spectrofluorometer (LS50B; PerkinElmer) using fluorescence intensity at 535 nm as described earlier [29].

### Measurement of cytosolic cytochrome c

B16F10 cells were treated with complex **3** (0, 2.5, 5, 10 and 25 μM) for 24 h, and the level of cytosolic cytochrome c was determined by a cytochrome c colorimetric assay kit, as described earlier [13].

### Confocal microscopy for Immunocytochemistry studies

B16F10 cells were treated with complex **3** (0 and 10 μM) for 24 h and confocal microscopy for immunocytochemistry analysis of p53 and p21 was done as reported earlier [13]. Cells were observed under an Andor spinning disk confocal microscope and images were acquired.

### Western blot analysis of protein expression in B16F10 cells following treatment with complex 3

Western blotting of the lysates of the cells treated with complex **3** (0, 2.5, 5 and 10 μM) for 24 h was performed using 10–15% SDS-PAGE gels, primary antibodies, alkaline phosphatase conjugated secondary antibodies and NBT-BCIP as a chromogenic substrate as described earlier [30]. The experiments were done for the detection of PARP cleavage and for determining expression levels of pro-caspase 3 and pro-caspase 9 along with cytosolic cytochrome c and Bax. The expressions of p53, p-p53 (ser 15), p21 and Bax were determined in cells treated with complex **3** (0, 5 and 10 μM) for 24 h. In another set of experiments, cells were pretreated with 15 and 30 μM of the p53 transactivation inhibitor pifthrin-α (PFT–α), then treated with complex **3** (10 μM) for 24 h and analyzed for the level of expressions of p53, p21, Bax and cytochrome c. MTT assay was conducted to determine the growth inhibitory action of complex **3** (0 and 10 μM) in presence of PFT-α (0, 5, 15 and 30 μM). Also cells treated with NAC (0, 5 and 10 mM) for 1 h and then treated with complex **3** (10 μM) for 24 h were analyzed for the level of p53 and p-p53 (ser 15) expression.

### Tumor Induction and evaluation of the antitumor activity of complex 3 in mice

In order to select the least toxic doses, male BALB/c mice were administered (i.p.) with 0, 5, 10, 20 and 40 mg/kg body weight of complex **3**, observed for 60 days, and inoculated with B16F10 cells as described earlier [31]. Briefly, mice inoculated with 5 × 10^4^ B16F10 cells in the right hind limb (subcutaneously) were monitored for 15 days, after which complex **3** (5 and 10 mg/kg mouse body weight) was administered by intraperitoneal (i.p.) injection. The final dose was given on the 23^rd^ day and the mice were sacrificed on the 24^th^ day. Care and maintenance of animals were done in adherence to the guidelines of the Institutional Animal Care and Use Committee.

After sacrifice, the tumors were excised out and H/E slides were prepared. Mitotic index of the tumors of mice administered with complex **3** (5 and 10 mg/kg of mice body weight) was counted with respect to the mice having the B16F10 cell induced tumor only. Along with tumor weight, tumor volume, body weight and the survival index of the mice were determined.

### Immunohistological analysis of the tumor proteins

Slides fixed in 4% paraformaldehyde were incubated with primary antibodies for p53, p21, NF-κB p65 and p50, VEGF and MMP-9 overnight at 4°C. After washing with 1X TBST, slides were incubated with secondary antibodies for the above proteins for 2 h and stained with DAPI for identification of the nucleus. Cells were observed under an Andor spinning disk confocal microscope and images were acquired.

### Statistical analysis

All the experiments were carried out in triplicate and values were reported as mean ± SD. Student’s *t* test was used for determining statistical significance (P < 0.05, P < 0.01 and P < 0.001). The survival rate data were analyzed by Kaplan-Meier plots.

## Competing interests

The authors declare that they have no competing interests.

## Authors’ contributions

Conceived and designed the experiments: AN, SKD and KDS planned, executed the experiments, analyzed the data and prepared the manuscript. SD and RNM contributed to in-vivo works. JD performed the chemistry works. KDS contributed reagents/materials/analysis tools. All authors read and approved the final manuscript.

## Supplementary Material

Additional file 1: Figure S1 FAB Mass spectroscopic data for Schiff base, **1**. Click here for file

Additional file 2: Figure S2 NMR data for the proligand, **2**. Click here for file

Additional file 3: Figure S3 FAB Mass spectroscopic data for the proligand, **3**. Click here for file

Additional file 4: Figure S4 NMR data for the gold (I) N-Heterocyclic complex, **3**. Click here for file

Additional file 5: Figure S5 FAB Mass spectroscopic data for the gold (I) N-Heterocyclic complex, **3**. Click here for file

Additional file 6: Table S1 GI_50_ of cancer cells in presence of cisplatin and complex **3** after 24 h. **Table S2.** Growth inhibition of cancer cells in presence of complex **3** (GI_50_ concentration) in a time dependent manner. **Table S3.** GI_50_ of cells in presence of cisplatin and complex **3** after 24 h. Click here for file

Additional file 7: Figure S6 Fluorescence Intensity graph for the expression of p53 and p21 in the presence of complex **3** (0 and 10 μM) after 24 h. Values are mean ± S.D and represent one of the 3 representative experiments. *P < 0.05. Click here for file

Additional file 8: Figure S7 Fluorescence Intensity graph for the expression of (A) p53 and p21 (B) NF-қB p65 and p50 subunits (C) VEGF and MMP-9 proteins in the presence of complex **3** (0 and 10 mg/kg body weight of mice). Values are mean ± S.D and represent one of the 3 representative experiments. *P < 0.05 and **P < 0.01. Click here for file

## References

[B1] Berners-Price SJ Activating platinum anticancer complexes with visible light Angew Chem Int Ed Engl 2011 50 804 805 10.1002/anie.201004552 21246674

[B2] Liu W Gust R Metal N-heterocyclic carbene complexes as potential antitumor metallodrugs Chem Soc Rev 2013 42 755 773 10.1039/c2cs35314h 23147001

[B3] Khramov DM Vincent ML Bielawski CW N-heterocyclic carbene-transition metal complexes: spectroscopic and crystallographic analyses of ð-back-bonding interactions Organometallics 2007 26 6042 6049 10.1021/om700591z

[B4] Rubbiani R Kitanovic I Alborzinia H Can S Kitanovic A Onambele LA Stefanopoulou M Geldmacher Y Sheldrick WS Wolber G Prokop A Wölfl S Ott I Benzimidazol-2-ylidene gold (I) complexes are thioredoxin reductase inhibitors with multiple antitumor properties J Med Chem 2010 53 8608 8618 10.1021/jm100801e 21082862

[B5] Melaiye A Simons RS Milsted A Pingitore F Wesdemiotis C Tessier CA Youngs WJ Formation of water-soluble pincer silver (I)-carbene complexes: a novel antimicrobial agent J Med Chem 2004 47 973 977 10.1021/jm030262m 14761198

[B6] Hickey JL Ruhayel RA Barnard PJ Baker MV Berners-Price SJ Filipovska A Mitochondria-targeted chemotherapeutics: the rational design of gold (I) N-heterocyclic carbene complexes that are selectively toxic to cancer cells and target protein selenols in preference to thiols J Am Chem Soc 2008 130 12570 12571 10.1021/ja804027j 18729360

[B7] Oehninger L Rubbiani R Ingo O N-Heterocyclic carbene metal complexes in medicinal chemistry Dalton Trans 2013 42 3269 3284 10.1039/c2dt32617e 23223752

[B8] Ingo O On the medicinal chemistry of gold complexes as anticancer drugs Coord Chem Rev 2009 253 1670 1681 10.1016/j.ccr.2009.02.019

[B9] Kim NH Park HJ Oh MK Kim IS Antiproliferative effect of gold (I) compound auranofin through inhibition of STAT3 and telomerase activity in MDA-MB 231 human breast cancer cells BMB Rep 2013 46 59 64 10.5483/BMBRep.2013.46.1.123 23351386 PMC4133824

[B10] Shaw III CF: gold-based therapeutic agents Chem Rev 1999 99 2589 2600 10.1021/cr980431o 11749494

[B11] Hudnall TW Tennyson AG Bielawski CW A seven-membered N, N’-diamidocarbene Organometallics 2010 29 4569 4578 10.1021/om1007665

[B12] Liu W Bensdorf K Proetto M Abram U Hagenbach A Gust R NHC gold halide complexes derived from 4, 5-diarylimidazoles: synthesis, structural analysis, and pharmacological investigations as potential antitumor agents J Med Chem 2011 54 8605 8615 10.1021/jm201156x 22091836

[B13] Dey SK Bose D Hazra A Naskar S Nandy A Munda RN Das S Chatterjee N Mondal NB Banerjee S Saha KD Cytotoxic activity and apoptosis-inducing potential of di-spiropyrrolidino and di-spiropyrrolizidino oxindole andrographolide derivatives PLoS One 2013 8 e58055 10.1371/journal.pone.0058055 23472133 PMC3589478

[B14] Saha A Lee YC Zhang Z Chandra G Su SB Mukherjee AB Lack of an endogenous anti-inflammatory protein in mice enhances colonization of B16F10 melanoma cells in the lungs J Biol Chem 2010 285 10822 10831 10.1074/jbc.M109.083550 20118237 PMC2856288

[B15] Kim HN Kim H Kong JM Bae S Kim YS Lee N Cho BJ Lee SK Kim HR Hwang YI Kang JS Lee WJ Vitamin C down-regulates VEGF production in B16F10 murine melanoma cells via the suppression of p42/44 MAPK activation J Cell Biochem 2011 112 894 901 10.1002/jcb.22997 21328462

[B16] Adhikary SD Bose D Mitra P Saha KD Bertolasid V Dinda J Au(I)- and Pt(II)-N-heterocyclic carbene complexes with picoline functionalized benzimidazolin-2-ylidene ligands; synthesis, structures, electrochemistry and cytotoxicity studies New J Chem 2012 36 759 767 10.1039/c2nj20928d

[B17] Valles SL Benlloch M Rodriguez ML Mena S Pellicer JA Asensi M Obrador E Estrela JM Stress hormones promote growth of B16-F10 melanoma metastases: an interleukin 6- and glutathione-dependent mechanism J Transl Med 2013 11 1 14 10.1186/1479-5876-11-1 23517603 PMC3608962

[B18] Shavaleev NM Bell ZR Accorsi G Ward MD Syntheses and structures of mononuclear Re (CO) 3Cl (NN) ‘complex ligands’ with a pendant imino-pyridine binding site, and preparation of some heterodinuclear Re(I)-lanthanide(III) complexes Inorg Chim Acta 2003 351 159 166

[B19] Samanta T Rana BK Roymahapatra G Giri S Mitra P Pallepogu R Chattaraj PK Dinda J Synthesis, structure and theoretical studies of Hg (II)–NH carbene complex of annulated ligand pyridinyl[1,2-a]{2-pyridylimidazol}-3-ylidene hexaflurophosphate Inorg Chim Acta 2011 375 271 279 10.1016/j.ica.2011.05.017

[B20] Wang JMF Chen LYC Lin BJI Synthesis, structure, and spectroscopic properties of gold (I)-carbene complexes Organometallics 1999 18 1216 1223 10.1021/om980718b

[B21] Mallick S Pal BC Vedasiromoni JR Kumar D Saha KD Corchorusin-D directed apoptosis of K562 cells occurs through activation of mitochondrial and death receptor pathways and suppression of AKT/PKB pathway Cell Physiol Biochem 2012 30 915 926 10.1159/000341469 22965801

[B22] Pellei M Gandin V Marinelli M Marzano C Yousufuddin M Dias HV Santini C Synthesis and biological activity of ester- and amide-functionalized imidazolium salts and related water-soluble coinage metal N-heterocyclic carbene complexes Inorg Chem 2012 51 9873 9882 10.1021/ic3013188 22946642

[B23] Wang CH Shih WC Chang HC Kuo YY Hung WC Ong TG Li WS Preparation and characterization of amino-linked heterocyclic carbene palladium, gold, and silver complexes and their use as anticancer agents that act by triggering apoptotic cell death J Med Chem 2011 54 5245 5249 10.1021/jm101096x 21671598

[B24] Schuh E Pflüger C Citta A Folda A Rigobello MP Bindoli A Casini A Mohr F Gold (I) carbene complexes causing thioredoxin 1 and thioredoxin 2 oxidation as potential anticancer agents J Med Chem 2012 55 5518 5528 10.1021/jm300428v 22621714

[B25] Yan JJ Chow AL Leung CH Sun RW Ma DL Che CM Cyclometalated gold (III) complexes with N-heterocyclic carbene ligands as topoisomerase I poisons Chem Commun (Camb) 2010 46 3893 3895 10.1039/c001216e 20401423

[B26] Jeo KI Jeong JY Jue DM Thiol-reactive metal compounds inhibit NF-kappa B activation by blocking I kappa B kinase J Immunol 2000 164 5981 5989 10820281 10.4049/jimmunol.164.11.5981

[B27] Kim NH Lee MY Park SJ Choi JS Oh MK Kim IS Auranofin blocks interleukin-6 signalling by inhibiting phosphorylation of JAK1 and STAT3 Immunology 2007 122 607 614 10.1111/j.1365-2567.2007.02679.x 17645497 PMC2266044

[B28] Mosmann T Rapid colorimetric assay for cellular growth and survival: application to proliferation and cytotoxicity assays J Immunol Methods 1983 65 55 63 10.1016/0022-1759(83)90303-4 6606682

[B29] Ratha J Majumdar KN Mandal SK Bera R Sarkar C Saha B Mandal C Saha KD Bhadra R A sphingolipid rich lipid fraction isolated from attenuated *Leishmania donovani* promastigote induces apoptosis in mouse and human melanoma cells in vitro Mol Cell Biochem 2006 290 113 123 10.1007/s11010-006-9174-y 16718368

[B30] Majumdar KN Banerjee A Ratha J Mandal M Sarkar RN Saha KD Leishmanial lipid suppresses tumor necrosis factor alpha, interleukin-1beta, and nitric oxide production by adherent synovial fluid mononuclear cells in rheumatoid arthritis patients and induces apoptosis through the mitochondrial-mediated pathway Arthritis Rheum 2008 58 696 706 10.1002/art.23295 18311833

[B31] Das S Chatterjee N Bose D Dey SK Munda RN Nandy A Bera S Biswas SK Das KD Anticancer potential of 3-(arylideneamino)-2-phenylquinazoline-4(3H)-one derivatives Cell Physiol Biochem 2012 29 251 260 10.1159/000337606 22415094

