# Supplementary figures and images for: Gold (I) N-heterocyclic carbene complex inhibits mouse melanoma growth by p53 upregulation
Source: Mol Cancer. 2014 Mar 13;13:57. doi: 10.1186/1476-4598-13-57 (PMC4007776; doi:10.1186/1476-4598-13-57)

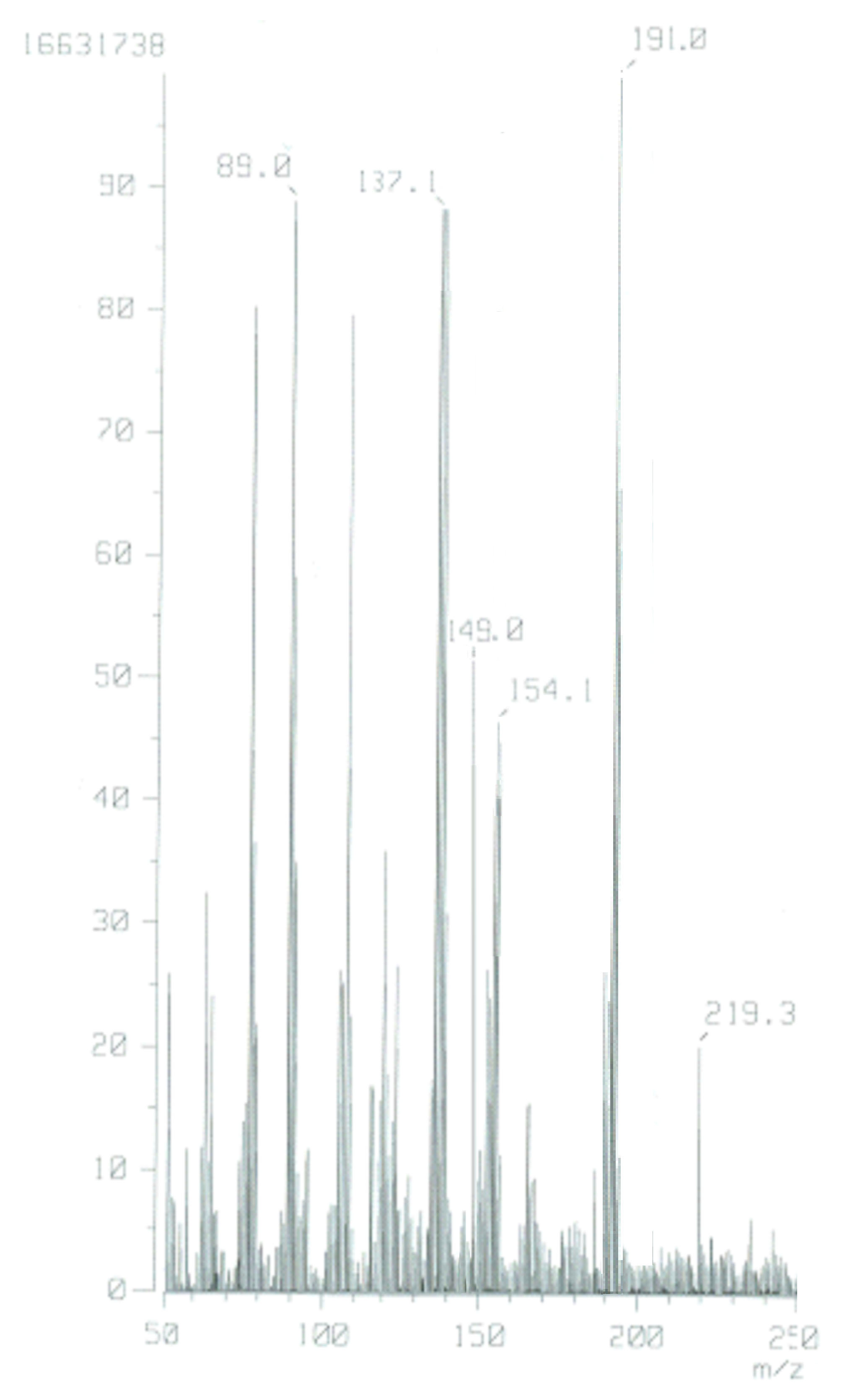

Supplement: Additional file 1: Figure S1 — FAB Mass spectroscopic data for Schiff base, 1. [file 1476-4598-13-57-S1.tiff]

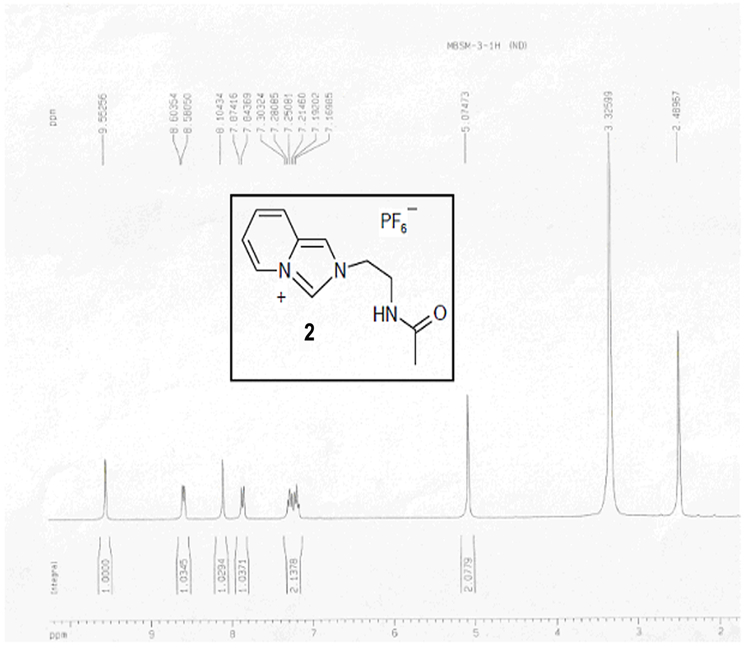

Supplement: Additional file 2: Figure S2 — NMR data for the proligand, 2. [file 1476-4598-13-57-S2.tiff]

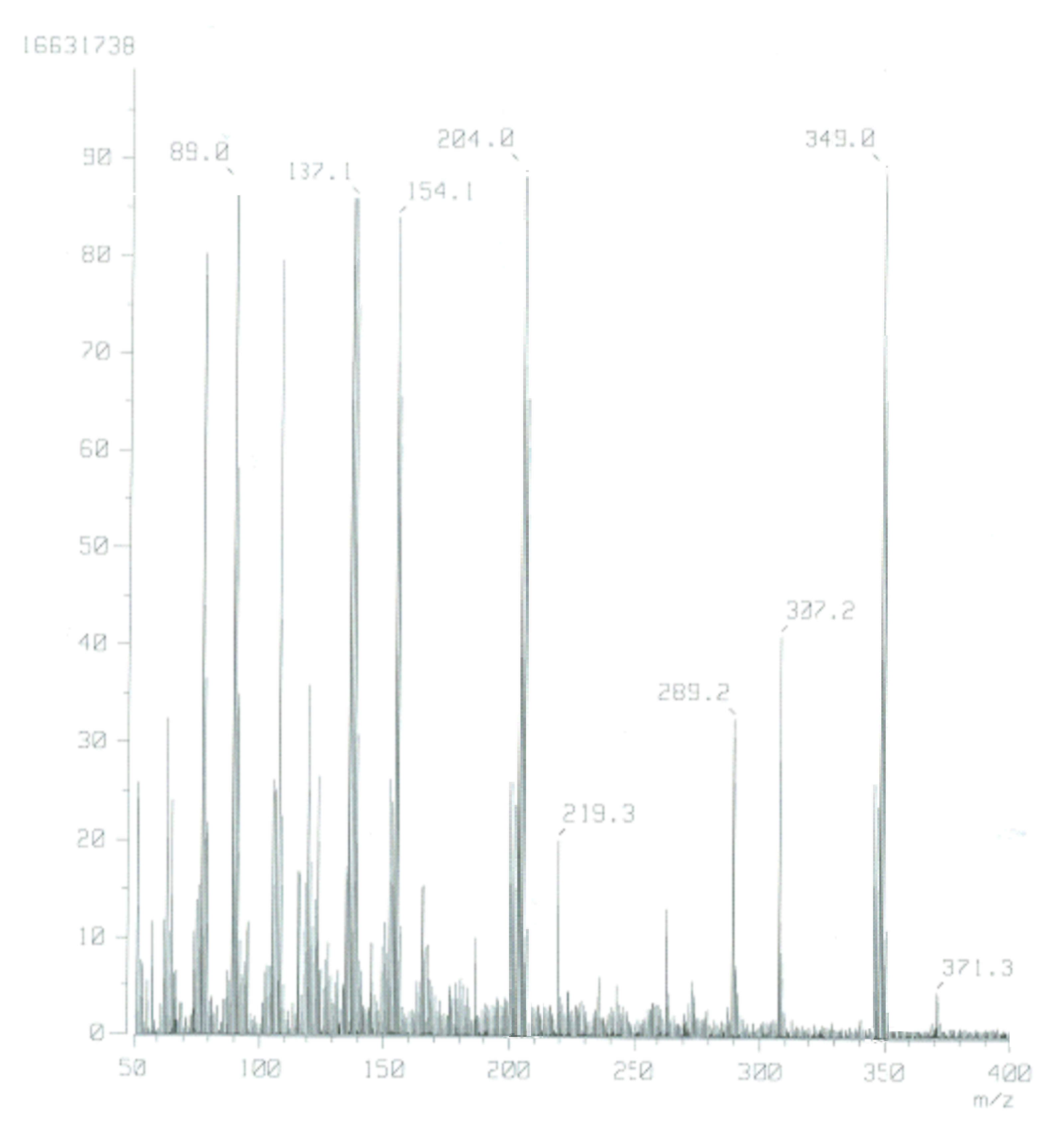

Supplement: Additional file 3: Figure S3 — FAB Mass spectroscopic data for the proligand, 3. [file 1476-4598-13-57-S3.tiff]

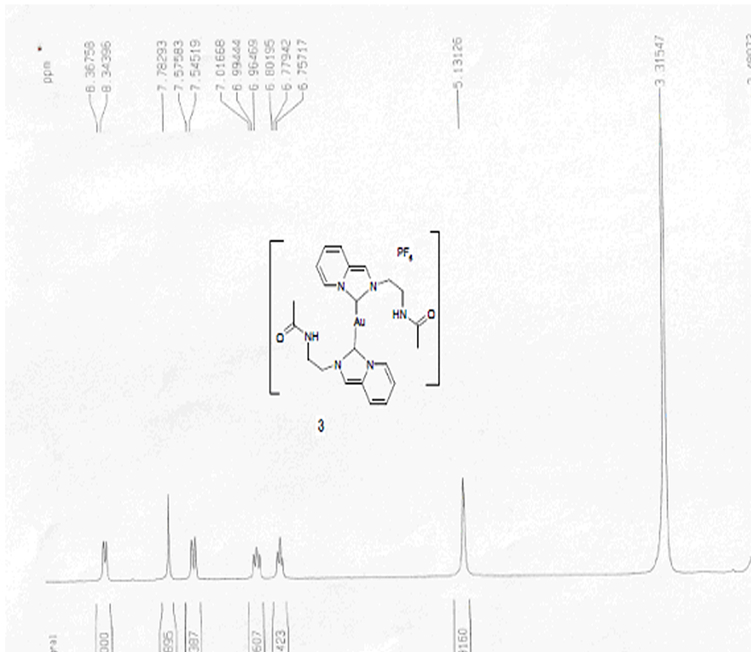

Supplement: Additional file 4: Figure S4 — NMR data for the gold (I) N-Heterocyclic complex, 3. [file 1476-4598-13-57-S4.tiff]

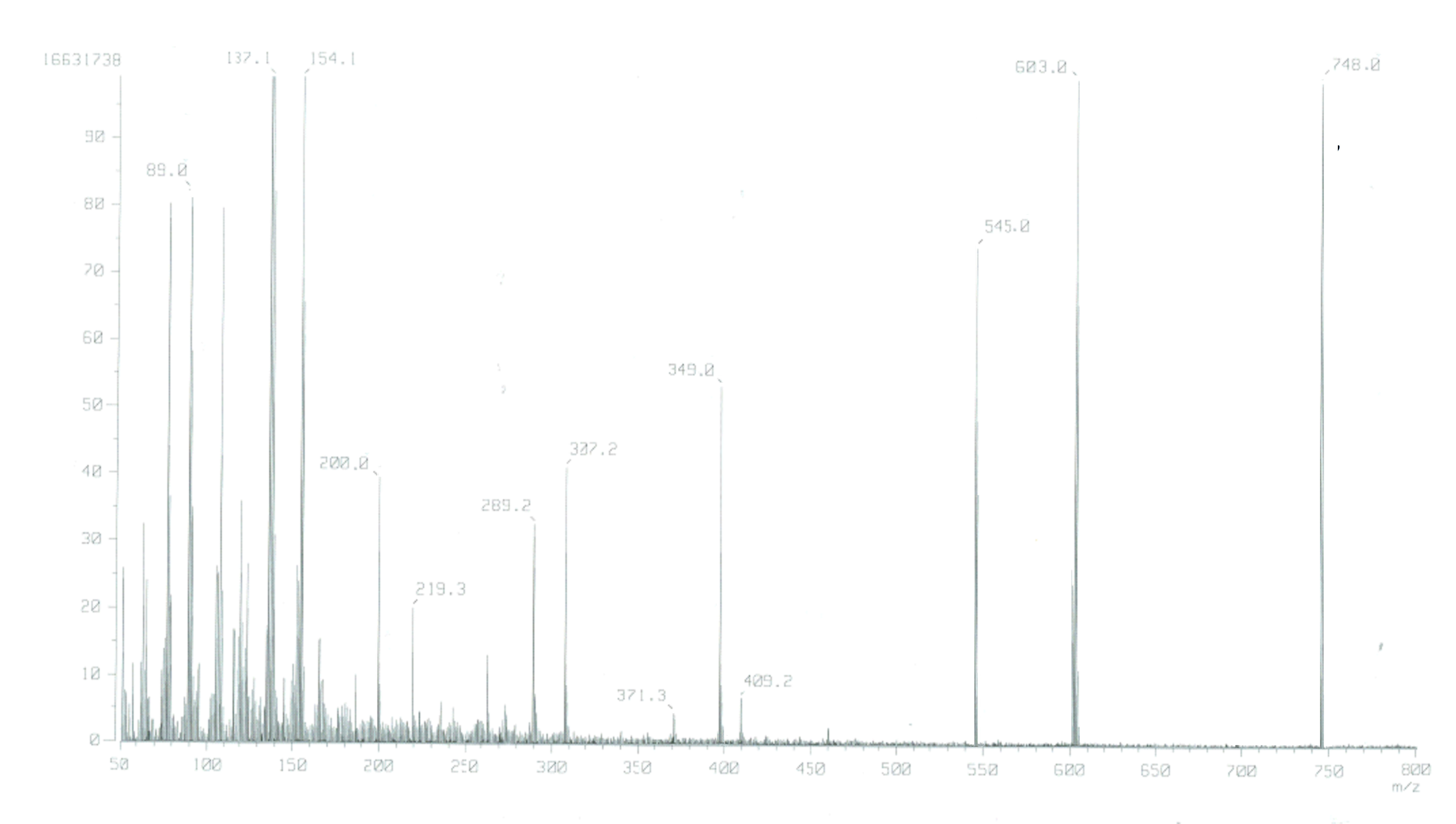

Supplement: Additional file 5: Figure S5 — FAB Mass spectroscopic data for the gold (I) N-Heterocyclic complex, 3. [file 1476-4598-13-57-S5.tiff]

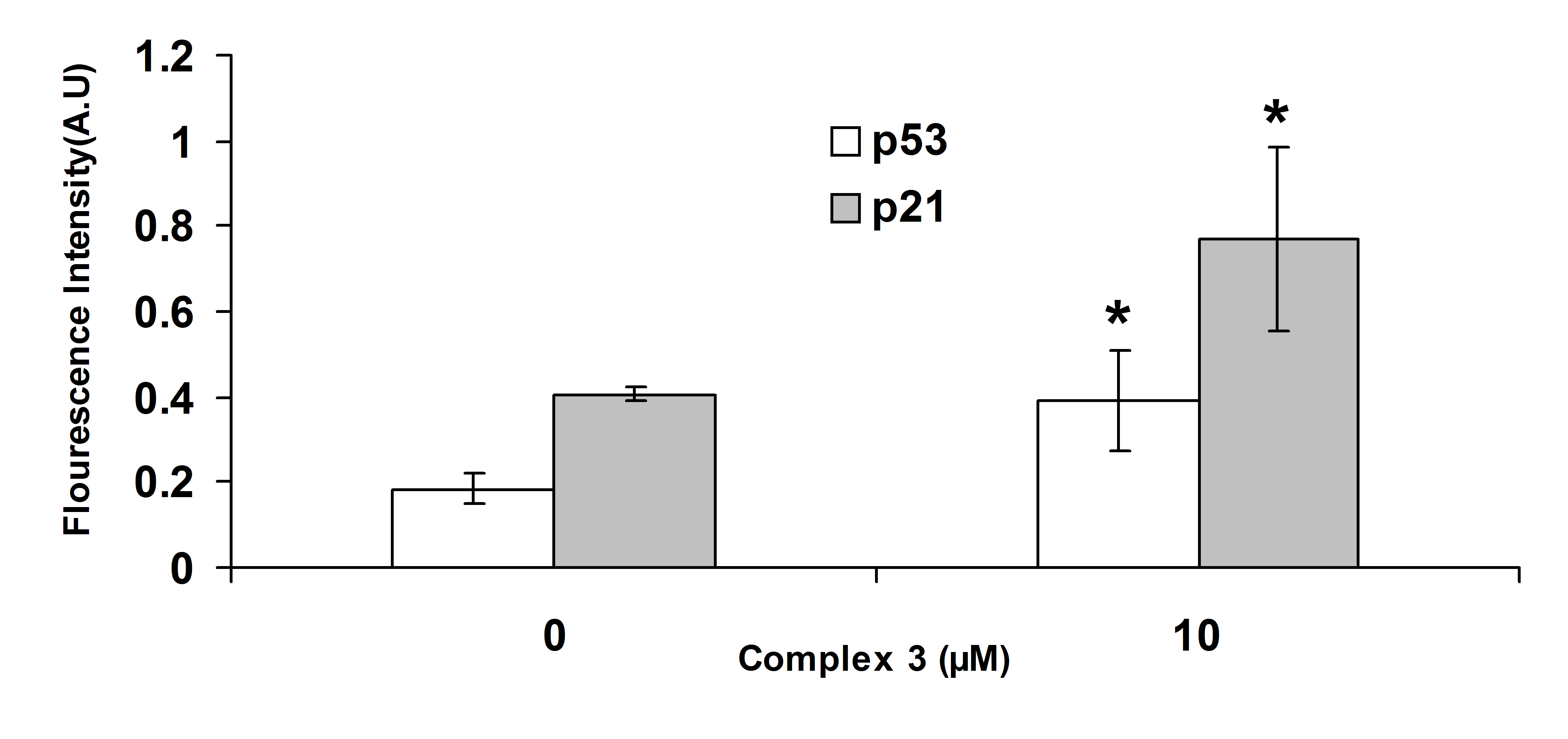

Supplement: Additional file 7: Figure S6 — Fluorescence Intensity graph for the expression of p53 and p21 in the presence of complex 3 (0 and 10 μM) after 24 h. Values are mean ± S.D and represent one of the 3 representative experiments. *P < 0.05. [file 1476-4598-13-57-S7.tiff]

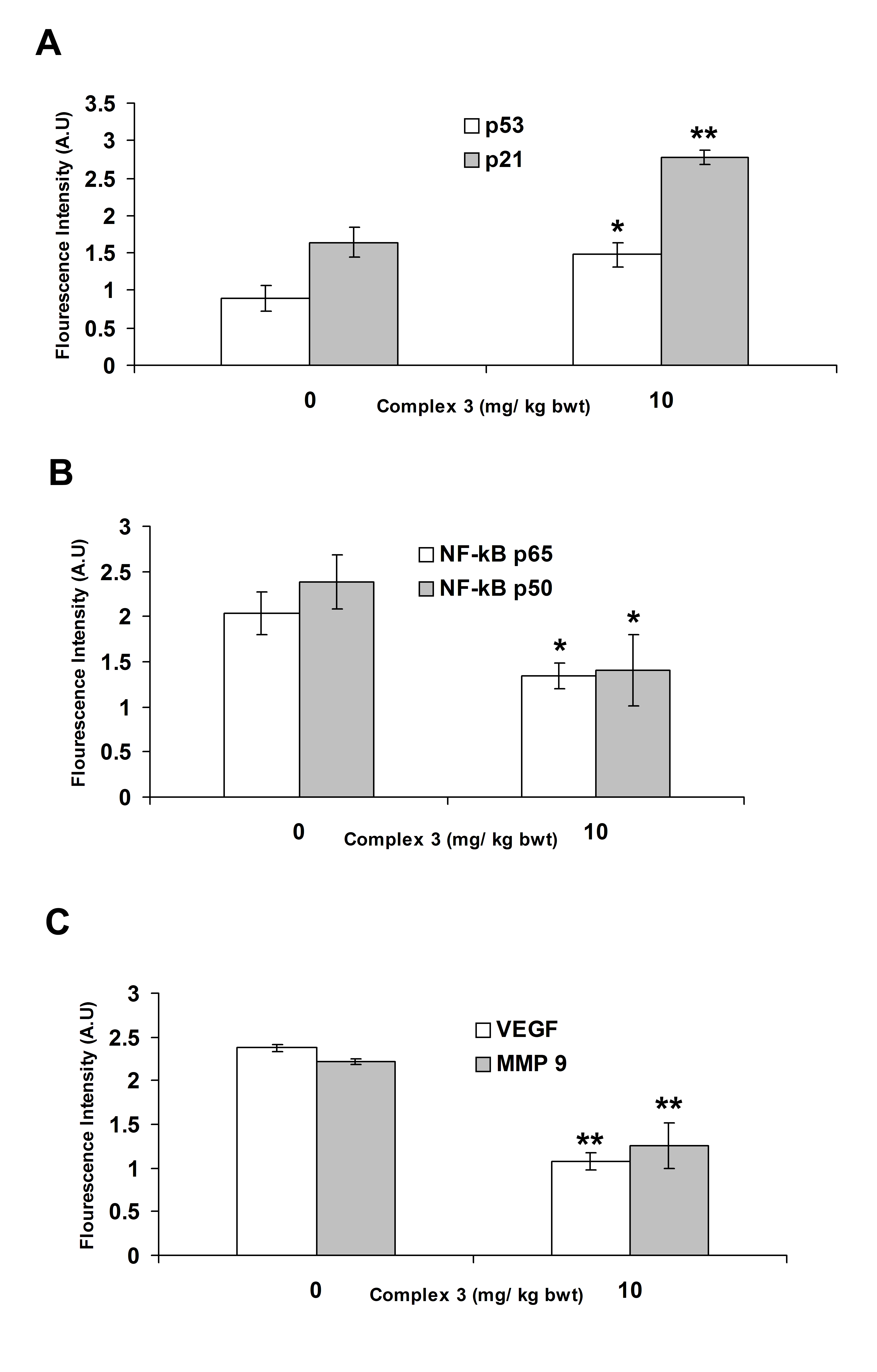

Supplement: Additional file 8: Figure S7 — Fluorescence Intensity graph for the expression of (A) p53 and p21 (B) NF-қB p65 and p50 subunits (C) VEGF and MMP-9 proteins in the presence of complex 3 (0 and 10 mg/kg body weight of mice). Values are mean ± S.D and represent one of the 3 representative experiments. *P < 0.05 and **P < 0.01. [file 1476-4598-13-57-S8.tiff]
