# Supplementary material for: Gold (I) N-heterocyclic carbene complex inhibits mouse melanoma growth by p53 upregulation
Source: Mol Cancer. 2014 Mar 13;13:57. doi: 10.1186/1476-4598-13-57 (PMC4007776; doi:10.1186/1476-4598-13-57)
Supplement: Additional file 6: Table S1 — GI50 of cancer cells in presence of cisplatin and complex 3 after 24 h. Table S2. Growth inhibition of cancer cells in presence of complex 3 (GI50 concentration) in a time dependent manner. Table S3. GI50 of cells in presence of cisplatin and complex 3 after 24 h. [file 1476-4598-13-57-S6.doc]

**Table S1.** GI_50_ of cancer cells in presence of cisplatin and complex **3** after 24 h.

| **Cells** | **Cisplatin (GI_50_)** | **Complex 3 (GI_50_)** |
| --- | --- | --- |
| HCT-116 | 4.80 ± 0.21 µM | 4.73 ± 0.20 µM |
| HepG2 | 8.50 ± 0.10 µM | 9.48 ± 0.25 µM |
| B16F10 | 5.00 ± 0.08 µM | 9.4 ± 0.26 µM |
| A549 | 6.80 ± 0.10 µM | 13.71 ± 0.15 µM |

Cells were treated with different concentration of cisplatin and complex **3** ranging from 0–50 µM for 24 h, respectively. GI_50_ values were calculated from MTT assay. Values are mean ± S.D and represent one of the 3 representative experiments. *P<0.05 and *P<0.01.

**Table S2.** Growth inhibition of cancer cells in presence of complex **3** (GI_50_ concentration) in a time dependent manner.

| **Cells** | **Complex 3 (12 h)** | **Complex 3 (24 h)** | **Complex 3 (36 h)** | **Complex 3 (48 h)** |
| --- | --- | --- | --- | --- |
| HCT-116 | 32 % | 55 % | 73 % | 77 % |
| HepG2 | 20 % | 48 % | 72 % | 75 % |
| A549 | 11 % | 42 % | 57 % | 60 % |
| B16F10 | 28 % | 51 % | 67 % | 67 % |

Cells were treated with GI_50_ concentrations complex **3** ranging for 12, 24, 36 and 48 h, respectively. Percentage of growth inhibition was calculated from MTT assay. Values are mean ± S.D and represent one of the 3 representative experiments. *P<0.05 and *P<0.01.

**Table S3.** GI_50_ of cells in presence of cisplatin and complex **3** after 24 h.

| **Cells** | **Cisplatin (µM)** | **Complex 3 (µM)** |
| --- | --- | --- |
| Peripheral blood mononuclear cells (PBMCs) | 10 | - 50 |

Cells were treated with different concentration of cisplatin and complex **3** ranging from 0–50 µM for 24 h, respectively. GI_50_ values were calculated from MTT assay. Values are mean ± S.D and represent one of the 3 representative experiments. *P<0.05 and *P<0.01.
